# Naringin attenuates *Actinobacillus pleuropneumoniae*-induced acute lung injury via MAPK/NF-κB and Keap1/Nrf2/HO-1 pathway

**DOI:** 10.1186/s12917-024-04055-2

**Published:** 2024-05-17

**Authors:** Qi-Lin Huang, Li-Na Huang, Guan-Yu Zhao, Chen Liu, Xiang-Yi Pan, Zhao-Rong Li, Xiao-Han Jing, Zheng-Ying Qiu, Rui-Hua Xin

**Affiliations:** 1 Lanzhou Institute of Husbandry and Pharmaceutical Sciences of Chinese Academy of Agricultural Sciences (CAAS), Lanzhou Gansu, 730000 China; 2 Engineering and Technology Research Center of Traditional Chinese Veterinary Medicine of Gansu Province, Lanzhou Gansu, 730000 China; 3 Key Laboratory of Veterinary Pharmaceutical Development of Ministry of Agriculture and Rural Affairs of P.R, Lanzhou Gansu, 730000 China; 4 School of Pharmacy, State Key Laboratory of Applied Organic Chemistry, Lanzhou University, Lanzhou Gansu, 730000 China; 5 College of Pharmacy, Gansu University of Chinese Medicine, Lanzhou Gansu, 730000 China

**Keywords:** *Actinobacillus pleuropneumoniae* (APP), Naringin (NAR), Anti-inflammatory mechanism, Antioxidant mechanism

## Abstract

*Actinobacillus pleuropneumoniae (APP)* causes porcine pleuropneumonia (PCP), which is clinically characterized by acute hemorrhagic, necrotizing pneumonia, and chronic fibrinous pneumonia. Although many measures have been taken to prevent the disease, prevention and control of the disease are becoming increasingly difficult due to the abundance of APP sera, weak vaccine cross-protection, and increasing antibiotic resistance in APP. Therefore, there is an urgent need to develop novel drugs against APP infection to prevent the spread of APP. Naringin (NAR) has been reported to have an excellent therapeutic effect on pulmonary diseases, but its therapeutic effect on lung injury caused by APP is not apparent. Our research has shown that NAR was able to alleviate APP-induced weight loss and quantity of food taken and reduce the number of WBCs and NEs in peripheral blood in mice; pathological tissue sections showed that NAR was able to prevent and control APP-induced pathological lung injury effectively; based on the establishment of an in vivo/in vitro model of APP inflammation, it was found that NAR was able to play an anti-inflammatory role through inhibiting the MAPK/NF-κB signaling pathway and exerting anti-inflammatory effects; additionally, NAR activating the Nrf2 signalling pathway, increasing the secretion of antioxidant enzymes Nqo1, CAT, and SOD1, inhibiting the secretion of oxidative damage factors NOS2 and COX2, and enhancing the antioxidant stress ability, thus playing an antioxidant role. In summary, NAR can relieve severe lung injury caused by APP by reducing excessive inflammatory response and improving antioxidant capacity.

**Supplementary Information:**

The online version contains supplementary material available at 10.1186/s12917-024-04055-2.

## Introduction

*Actinobacillus pleuropneumoniae* (APP) can cause porcine pleuropneumonia (PCP), which mainly invades the tonsils and upper respiratory tract of pigs and colonizes and multiplies in the lungs. After infection with APP, acute clinical cases are characterized by acute hemorrhagic, necrotizing pneumonia [1], and chronic issues are characterized by localized necrosis of the lungs and chronic fibrinous pleuropneumonia [2]. APP transmission is predominantly airborne [3] and is highly contagious. APP spreads worldwide due to frequent international introductions and is susceptible to more harmful mixed infections with swine influenza, *Mycoplasma pneumoniae*, *Pasteurella multocida*, etc [4]. There are many APP serovars [5]; hence, vaccine cross-protection is weak. More importantly, APP bacterial resistance is increasing due to the inappropriate use of antibiotics in the farming focus [6, 7], making its pharmacological control much more complicated [8], causing severe economic losses to the farming industry. Therefore, there is an urgent need to target the development of novel drugs capable of combating APP infections.

In the pericarp of the rutaceae fruit, NAR can account for up to 98% of the total flavonoids [9], while in the fruit, NAR can account for more than 50% of the total flavonoids [10]. NAR has been reported to have a significant therapeutic effect on respiratory diseases, For instance, NAR could inhibit airway inflammation and improves lung endothelial hyperpermeability by upregulating Aquaporin1 in LPS/cigarette smoke-induced mice [11]. NAR can not only reduce the lung injury induced by COVID-19 by inhibiting interleukin-6 (IL-6) [12], but also block LPS-induced pulmonary edema by inhibiting the secretion of MPO, tumor necrosis factor-α (TNF-α) and neutrophil infiltration [13]. However, the efficacy and mechanism of NAR on APP-induced lung inflammation have not been reported. In our previous study, we found that NAR could alleviate APP-induced inflammatory response by inhibiting the signalling activation mechanism of NLRP3 inflammasome [14]. Therefore, in this experiment, We hope to explore whether NAR has multi-targeting in anti-APP from the perspective of anti-inflammatory and antioxidant.

Porcine alveolar macrophages (PAMs), as “sentinels” of the body’s immune system, play an essential role in the maintenance of immune homeostasis in the porcine lung and host defense [15]. When APP enters the organism, the virulence factors it carries first stimulate the Toll-like receptor 4 (TLR4) receptor on the PAMs, activating the MAPK/NF-κB signaling pathway, releasing excessive inflammatory factors, and inhibiting the secretion of anti-inflammatory factors [16, 17]; when inflammatory factors are overloaded in lung tissues, oxidative stress injury is induced [18], resulting in elevated peripheral blood neutrophils (NEs), white blood cells (WBCs) and C-reactive protein (CRP), as well as the inflammatory injury of lung tissues caused by pulmonary edema and infiltration of inflammatory factors [19]. Damaged lung tissues further provide opportunities for APP invasion, allowing the immune system to be over-activated, exacerbating inflammatory responses and oxidative stress, leading to severe lung tissue injury. Therefore, inhibition of excessive inflammatory and oxidative stress responses is the key to alleviate lung tissue injury. Therefore, therefore, in this experiment, we used APP to construct a mouse pneumonia model and an inflammatory cell model to explore the protective mechanism of NAR on lung tissue in terms of anti-inflammation and anti-oxidation.

## Materials and methods

### Chemicals and reagents

Naringin (NAR, purity of 98%) was purchased from Sigma Aldrich Chemical (St. Louis, MO, USA), RPMI 1640 medium (Gibco) and trypsin was purchased from JS Biosciences Co., Ltd (Lanzhou City, Gansu Province, China), fetal bovine serum (FBS) and penicillin-streptomycin antibiotics were purchased from Thermo Fisher Scientific (Massachusetts, USA), 3-(4,5-dimethylthiazol-2-yl)-2,5-diphenyl tetrazolium bromide (MTT) kit was purchased from Labgic Technology Co., Ltd. (Beijing, China), RNA extraction kit, TRIzol® Reagent RT- RNA extraction kit, TRIzol® Reagent RT- PCR kit and SYBR® green PCR master mix was purchased from TaKaRa (Tokyo, Japan), TLR4(Cell Signalling Technology, 14,358), p-IKKα/β(Cell Signalling Technology, 2697), IKKα(Cell Signalling Technology, 2682), IKKβ(Cell Signalling Technology, 8943), p-IkBα(Cell Signalling Technology, 2859), IkBα(Cell Signalling Technology, 2859), p-P38(proteintech, 28796-1-AP), P38(proteintech, 14064-1-AP), p-JNK(proteintech, 80024-1-RR), JNK(proteintech, 66210-1-Ig), p-ERK(proteintech, 28733-1-AP), ERK(proteintech, 11257-1-AP), Nrf2(proteintech, 80593-1-RR), Keap-1(proteintech, 80744-1-RR), HO-1(proteintech, 10701-1-AP), GAPDH(Cell Signalling Technology, 2118), Goat anti-Mouse IgG (H&L)-HRP(proteintech, PR30012), Goat anti-Rabbit IgG (H&L)-HRP(proteintech, PR30011), bovine serum albumin (BSA) and ECL assay kits were provided by Thermo Fisher Scientific (Massachusetts, USA). All other reagents used in the study were commercially available analytical-grade reagents unless otherwise stated.

### Bacteria, cellular inflammation models and NAR treatment

Porcine *Actinobacillus pleuropneumoniae* (APP) isolated from swine farms and verified by Blast sequencing was conserved in our laboratory and resuscitated in TSA medium (additionally supplemented with 5% fetal bovine serum and 1%NAD) at 37 °C. Porcine alveolar macrophages (PAMs) cells were purchased from Procell Life Science & Technology Co. After the cell fusion reached about 70–80%, the cells were digested with trypsin and passaged for culture. In the experiment, the cells were pretreated with different concentrations of NAR (10 µg/mL, 20 µg/mL, 30 µg/mL, and40µg/mL) for 12 h. Then, the cells were stimulated with APP bacteriophage solution (1 × 108CFU/mL) for 1 h. After removing the bacteriophage solution, the cells were cultured as usual for 12 h. The cells were collected for Western blotting and RT-PCR assays, and the supernatant was collected for ELISA.

### Test animals and treatments

Forty SPF-grade KM male mice weighing 18–20 g were purchased from Lanzhou Veterinary Research Institute (Licence No. SCXK Gansu 2023-016). All mice were housed under standard conditions (12 h/12 h light/dark cycle, 22–25 °C) in ventilated racks with an automatic watering system and fed ad libitum with standard chow. The mice were randomly divided into five groups, namely the control group (Control), the model group (Model), the NAR low-dose group (NARL, 20 mg/kg), the NAR medium-dose group (NARM, 40 mg/kg), and the NAR high-dose group (NARH, 80 mg/kg). The drugs were administered by gavage continuously for 8 d. Saline was given to the control and model groups. On 7 d, mice were anesthetized by intraperitoneal injection of sodium pentobarbital (50 mg/kg b.w.) and infected with APP utilizing tracheal intubation (100 µL each, bacterial concentration of 1 × 108 CFU/mL). The control group was injected with an equal volume of sterile saline. The daily number of deaths, body weight, and feed intake of the animals were recorded. The mice in each group were anaesthetized and executed 48 h after successful modeling, and different samples were taken for the following steps.

### Lung wet/dry weight ratio (W/D)

Lung W/D assessed the severity of APP-induced pulmonary edema in mice. The specific method was to remove the lungs of each group of mice, weigh them (wet weight of the lungs), and dry them in an oven at 70℃ until a constant weight (dry weight of the lungs) was obtained, and the W/D ratio was calculated.

1 $$W/D = wet\,lung\,weight\,(g)/drylungweight\,(g)$$

### Blood analysis

In each group of mice, the periocular hair was clipped with scissors, and the eyeballs were punctured to obtain blood. The removed blood was analyzed for WBCs and NEs in the peripheral blood by the Abaxis Blood Chemistry Analyser.

### Histopathologic examination

Fresh mouse lung tissues were fixed in 4% paraformaldehyde for 3 d, dehydrated, and embedded in paraffin wax. The wax blocks were cut into 5 μm-thick sections with a microtome, stained with hematoxylin-eosin (H&E) and Masson staining, and finally sealed with neutral gum. The sections were placed under a microscope (DM 4000B, Leica, Germany) to observe the mouse lung tissue.

### Quantitative real-time PCR analysis

Total RNA was isolated from cells or lung tissues using TRIzol reagent, RNA was reverse transcribed into cDNA according to the PrimeScript RT Reverse Transcription Kit, and mRNA expression was quantified using the Applied Biosystems Real-Time Fluorescence Quantitative PCR System and the SYBR premix Ex Taq II. Quantification results were calculated using the 2^-ΔΔCT^ method for comparison with β-actin as the reference mRNA (Table 1).

**Table 1:** Primer sequences of the target genes

Target	Sequence (5′–3′)		Orientation
*TLR4* *TLR4*		5’-AAACCACTCCACTCCCTCAG-3’ 5’-CTTCTGGTCCTTGACCCACT-3	Forward Reverse
*IL-10* *IL-10*		5’-CTGAGAACAGCTGCATCCAC-3’ 5’-AAAGTCCTCCAGCAGAGACC-3’	Forward Reverse
*SOD1* *SOD1*		5’- ATCAAGAGAGGCACGTTGGA-3’ 5’- GGGCGATCACAGAATCTTCG − 3’	Forward Reverse
*CAT* *CAT*		5’- ACATGGTCTGGGACTTCTGG-3’ 5’- CATGTGCCTGTGTCCATCTG-3’	Forward Reverse
*Nqo1* *Nqo1*		5’- GCTTACACATACGCTGCCAT-3’ 5’- GCCACAGAAATGCAAAGTGC-3’	Forward Reverse
*COX2* *COX2*		5’- AAAGCCTTGCTGTTCCAACC-3’ 5’- TTGGAGTGGGCTTCAGGAAT-3’	Forward Reverse
*Nos2* *Nos2*		5’- GGGTCAGAGCTACCATCCTC-3’ 5’- CGTCCATGCAGAGAACCTTG-3’	Forward Reverse
*β-actin* *β-actin*		5’-GGTCACCAGGGCTGCTTT-3’ 5’-ACTGTGCCGTTGACCTTGC-3’	Forward Reverse

### Western blotting analysis

Aliquots of lung tissue or cell samples were collected and lysed, centrifuged at 4 °C, 3000 r/min for 10 min, and the supernatant was collected and added to RIPA lysate containing 1% inhibitor (Beyotime, Shanghai, China), and the total protein concentration of the samples was detected by the BCA method (Beyotime, Shanghai, China) to ensure the The total protein concentration of the samples was consistent. The samples were added into different gel wells for gel electrophoresis, and 1× SDS-PAGE buffer was poured into the electrophoresis tank until the Marker migrated to the lower edge of the gel by 1 cm to stop electrophoresis. Then it was transferred to a polyacrylonitrile cellulose (NC) membrane (Merck, USA). The 5% skimmed milk powder was sealed for 1 h, and then primary and secondary antibodies were added separately according to the corresponding incubation instructions. Detection of TLR4 (1-1000), p-IKKα/β (1:1000), IKKα (1:1000), IKKβ (1:1000), p-IkBα (1:1000), IkBα (1:1000), p-P38 (1:1000), P38 (1:1000), p-JNK (1:1000), JNK ( 1: p-ERK (1:1000), ERK (1:1000), Nrf2 (1:1000), Keap-1 (1:1000) and HO-1 (1:1000) protein expression levels were detected using GAPDH (1:1000) as an internal reference. Finally, the development process was performed, and after scanning, the well-exposed films were saved and analyzed in grayscale using Image J software.

### Statistics

Western blot images were processed using Image J and Image-pro plus 6.0. All experiments had at least three replicates, the data were expressed as “mean ± standard deviation (M ± SD),” and the data were first tested for normal distribution and chi-square. The One-Way ANOVA method was used for the comparison of the groups, and the non-parametric test was used for the comparison of the groups that did not meet the normal distribution. Multiple comparisons were conducted using ISD pre-test and SNK post-test. *P* < 0.05 was taken as the difference was statistically significant. SPSS 27 was used for statistical data analysis, and GraphPad Prism 9 was used for graphing.

## Results

### Effects of NAR on APP-induced pulmonary edema and peripheral blood in mice

**Fig. 1:**
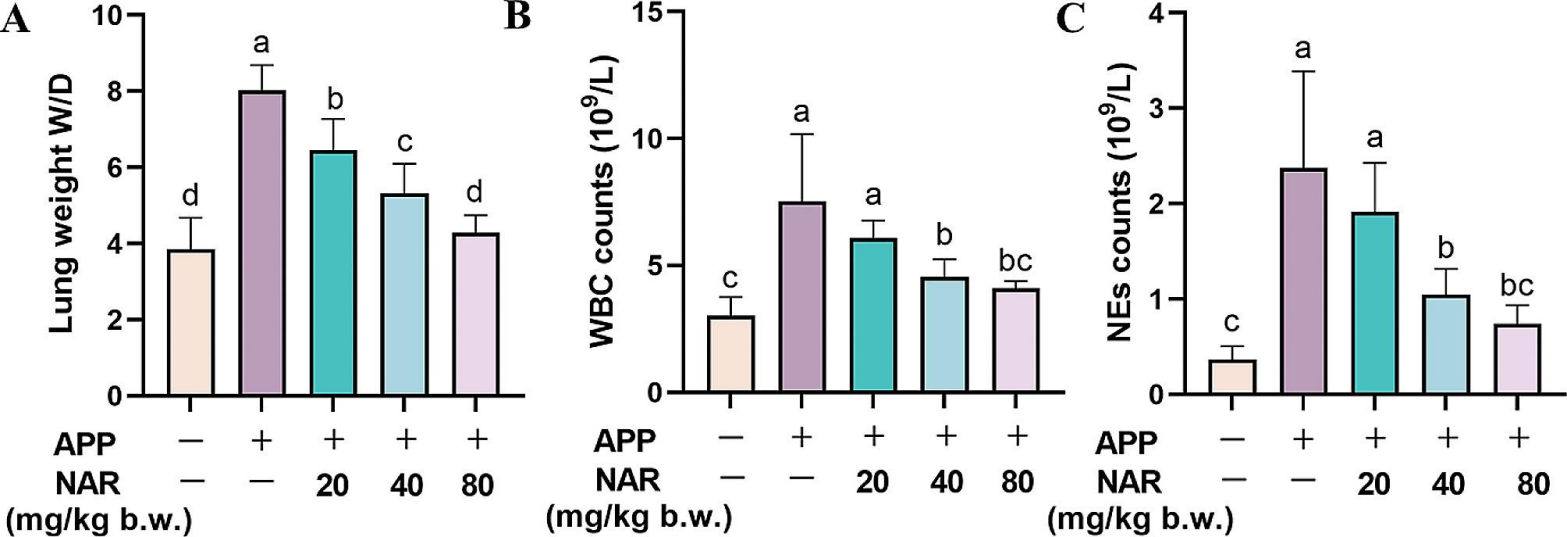
Effects of NAR on lesions in APP-induced mice. (A) Effects of NAR on W/D of lungs of mice, results are from one of three independent experiments (*n* = 3); (B-C) Effects of NAR on WBCs and NEs in peripheral blood of mice, results are from one of six independent experiments (*n* = 6). Different lowercase letters on the error bars indicate statistically significant differences (*P* < 0.05)

The ratio of wet weight to dry weight of lung tissue is an important evaluation index of pulmonary edema, we examined whether NAR could improve APP-induced lung edema by comparing the wet and dry weight ratios (W/D) of lung tissue in mice. We found that W/D was significantly higher in the APP-infected group than in the control group. In contrast, it was lower than that of the model group at all doses of NAR in a dose-dependent manner (Fig. 1A). When inflammatory injury occurs in the lungs, the levels of WBCs and NEs in peripheral blood are closely related to the severity of their lesions. Therefore, we collected peripheral blood from mice and found that WBCs and NEs were significantly elevated in the peripheral blood of mice in the model group. In contrast, NAR could dose-dependently inhibit the elevation of WBCs and NEs, thereby reducing lung injury (Fig. 1B-C). This is consistent with previous results in bronchoalveolar lavage fluid (BALF) [14].

### NAR alleviates APP-induced pathological lung injury in mice

**Fig. 2:**
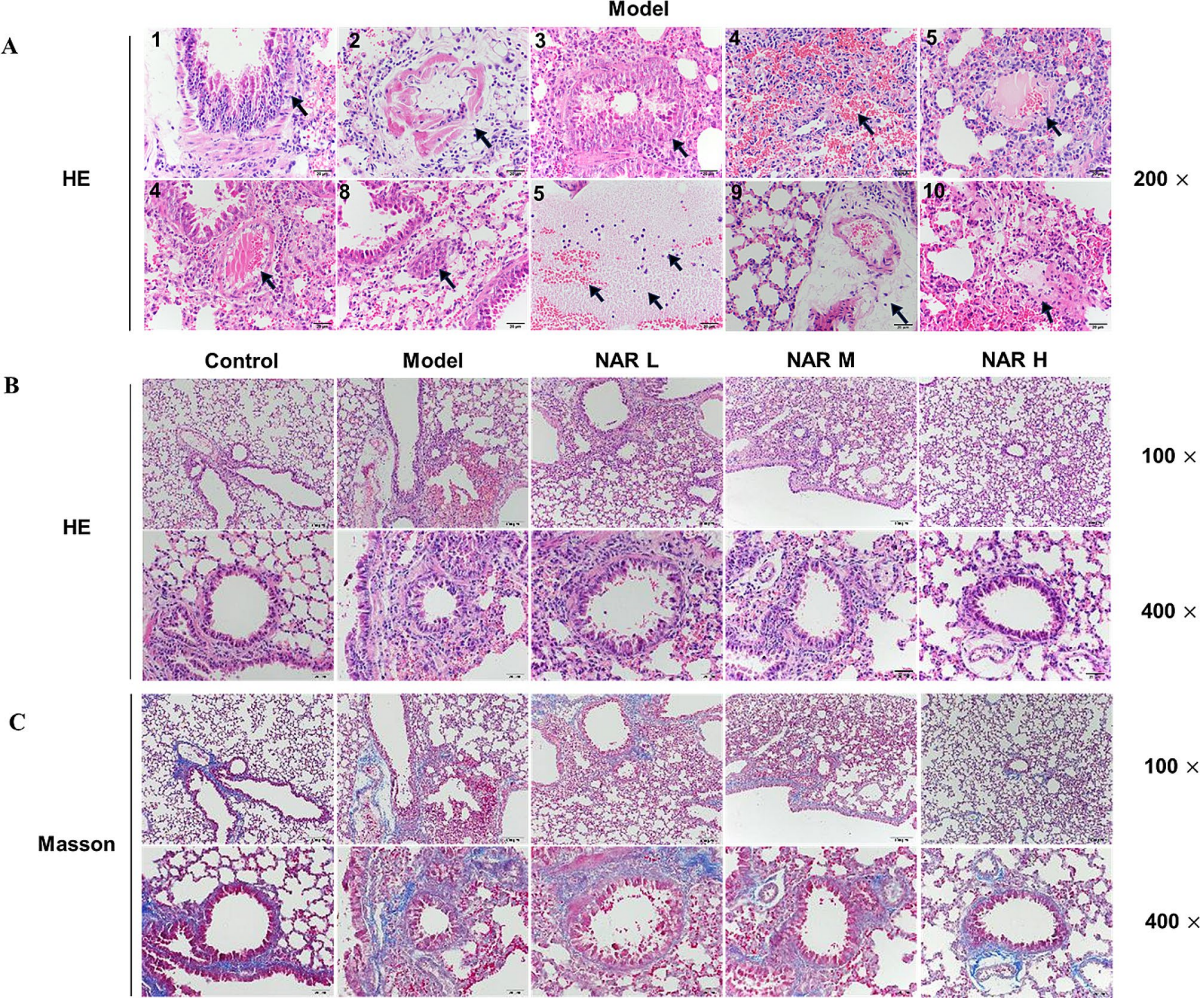
Effect of NAR on APP-induced histopathological damage in the lungs of mice. (A) Pathological damage in the lungs caused by APP infection; (B) Effect of NAR on APP-induced pathological damage in the lungs of mice; (C) Effect of NAR on the degree of APP-induced lung tissue fibrosis in the lungs of mice. Results are from one of three independent experiments (*n* = 3)

**Fig. 3:**
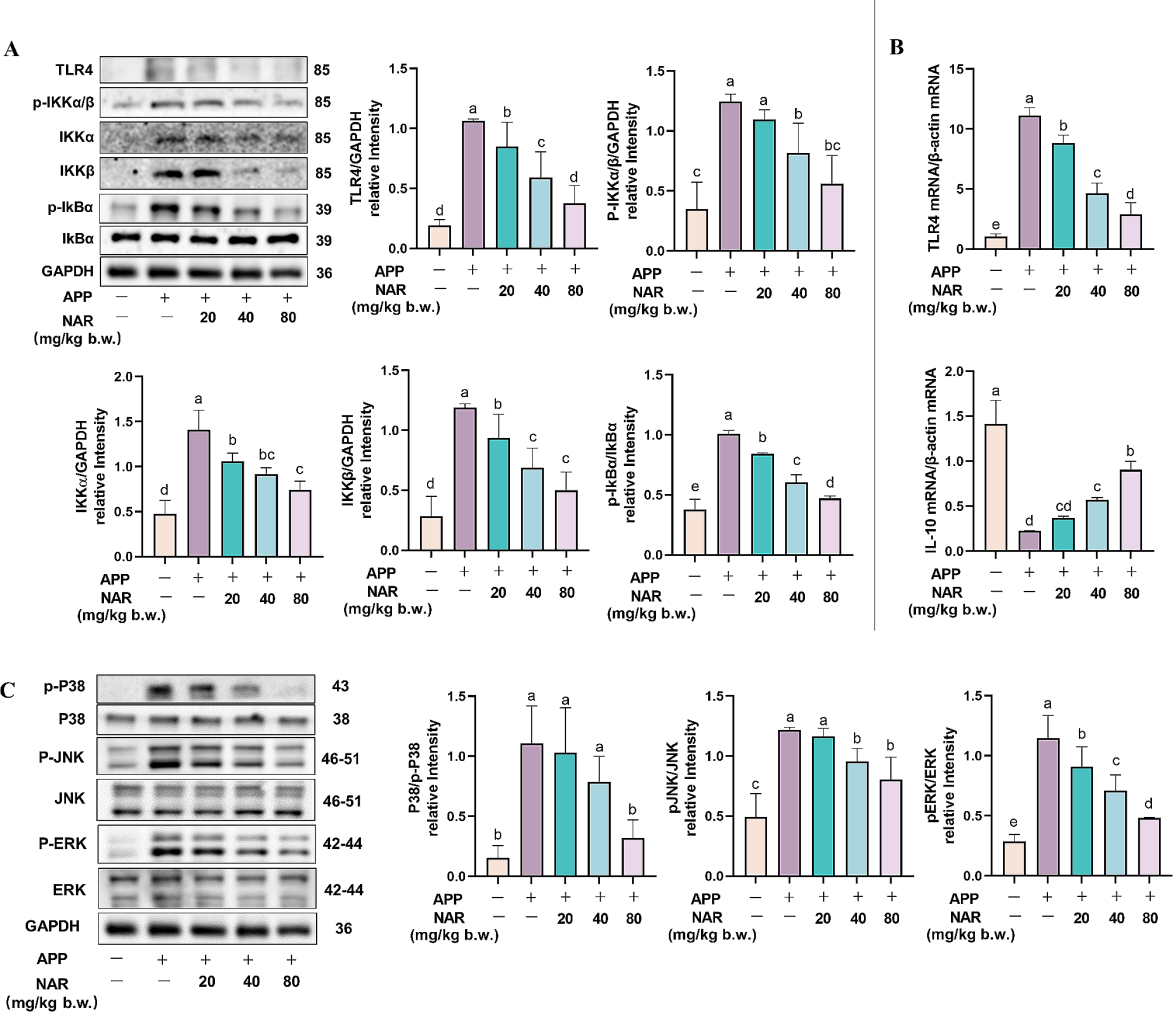
NAR exerts anti-inflammatory effects on APP mice. Effect of NAR on the expression of critical proteins in the NF-κB signalling pathway in APP mice; (B) Effect of NAR on the mRNA expression of TLR4 and IL-10 in the lungs of APP-induced mice; (C) Effect of NAR on the phosphorylation levels of P38, JNK and ERK proteins in the lungs of APP-induced mice. Different lowercase letters on the error bars indicate statistically significant differences (*P* < 0.05), results are from one of three independent experiments (*n* = 3)

By H & E staining, it was found that the alveolar ciliated columnar epithelium of the mice in the model group was degenerated and detached, some bronchial squamous epithelium was metaplasia (Fig. 2A 1), the walls of the vascular tubes and the pulmonary fine bronchioles were edematous, congested, and hyperplastic (Fig. 2A Figs. 2 and 3); The interstitial stroma of the lungs was seen to be diffusely thickened, edematous and haemorrhagic in the alveolar walls (Fig. 2A 4), and the alveolar lumens and capillaries were plasma-tinged exudate (Fig. 2A 5–6), with a large number of foam-like macrophages (Fig. 2A 7); at the same time, the lung tissues of mice also showed lesions typical of porcine APP, such as congestion of alveolar capillaries with large numbers of erythrocytes, leukocytes, and NEs as well as fibrinous thrombus formation in the blood vessels (Fig. 2A 8), a small number of inflammatory cells in the alveolar luminal fibrinoid network(Fig. 2A 9), and fibrinous pneumonia (Fig. 2A 10), with a large amount of proliferation of connective tissues. After NAR administration, there were different degrees of relief of lung tissue injury in each dose group compared with the model group, the trachea was more structurally intact and the epithelial cells of the tracheal mucosa were better aligned. In addition, after the intervention of APP, the inflammatory exudate filling in the fine bronchi disappeared, and the infiltration of peripheral inflammatory cells was reduced (Fig. 2B). Masson staining found a large amount of sparse edema and fibrosis formation around the blood vessels and bronchioles in the model group of mice compared with the control group (Fig. 2C). In contrast, the degree of pulmonary fibrosis in mice in all APP administration groups was reduced in a dose-dependent manner.

### NAR inhibits APP-induced pneumonia in mice by modulating the MAPK/NF-κB signalling pathway

NF-κB is a classical inflammatory pathway; to investigate the mechanism of action of NAR to inhibit inflammation for the treatment of PCP, we further examined the critical proteins in the NF-κB signalling pathway and found that the protein expression of TLR4, p-IKKα/β, IKKα, IKKβ, and p-IkBα was significantly increased in the lung tissues of the mice in the model group (*P <* 0.05) and that NAR was able to reduce the protein expression of TLR4, p-IKKα/β, IKKα, IKKβ, and p-IkBα (Fig. 3A). Meanwhile, we examined the TLR4 and IL-10 mRNA content in lung tissues. We found that APP significantly promoted TLR4 mRNA expression and reduced mRNA expression of anti-inflammatory factor IL-10 compared to the control group (*P <* 0.05). NAR intervention reduced the gene expression of TLR4 and IL-10 and alleviated the imbalance of inflammatory factor secretion caused by APP (Fig. 3B). The mitogen-activated protein kinase family (MAPK) is essential as a key regulator of inflammation and plays a critical role in the development of inflammation. To deeply investigate the anti-inflammatory mechanism of NAR in APP infection, the protein expression levels of ERK, JNK, and P38, three crucial members of the MAPK pathway, were detected in mouse lung tissues by Western blot. The results showed that the phosphorylation levels of P38, JNK, and ERK were significantly increased after APP infection compared with the control group. At the same time, NAR interference significantly reduced the phosphorylation levels of these three but did not affect the total ERK, JNK, and P38 protein levels (Fig. 3C). In summary, NAR can exert anti-inflammatory effects on APP-induced pneumonia mice by inhibiting the MAPK/NF-κB signalling pathway.

### NAR inhibits APP-induced pneumonia in mice by activating the Nrf2 signalling pathway

**Fig. 4:**
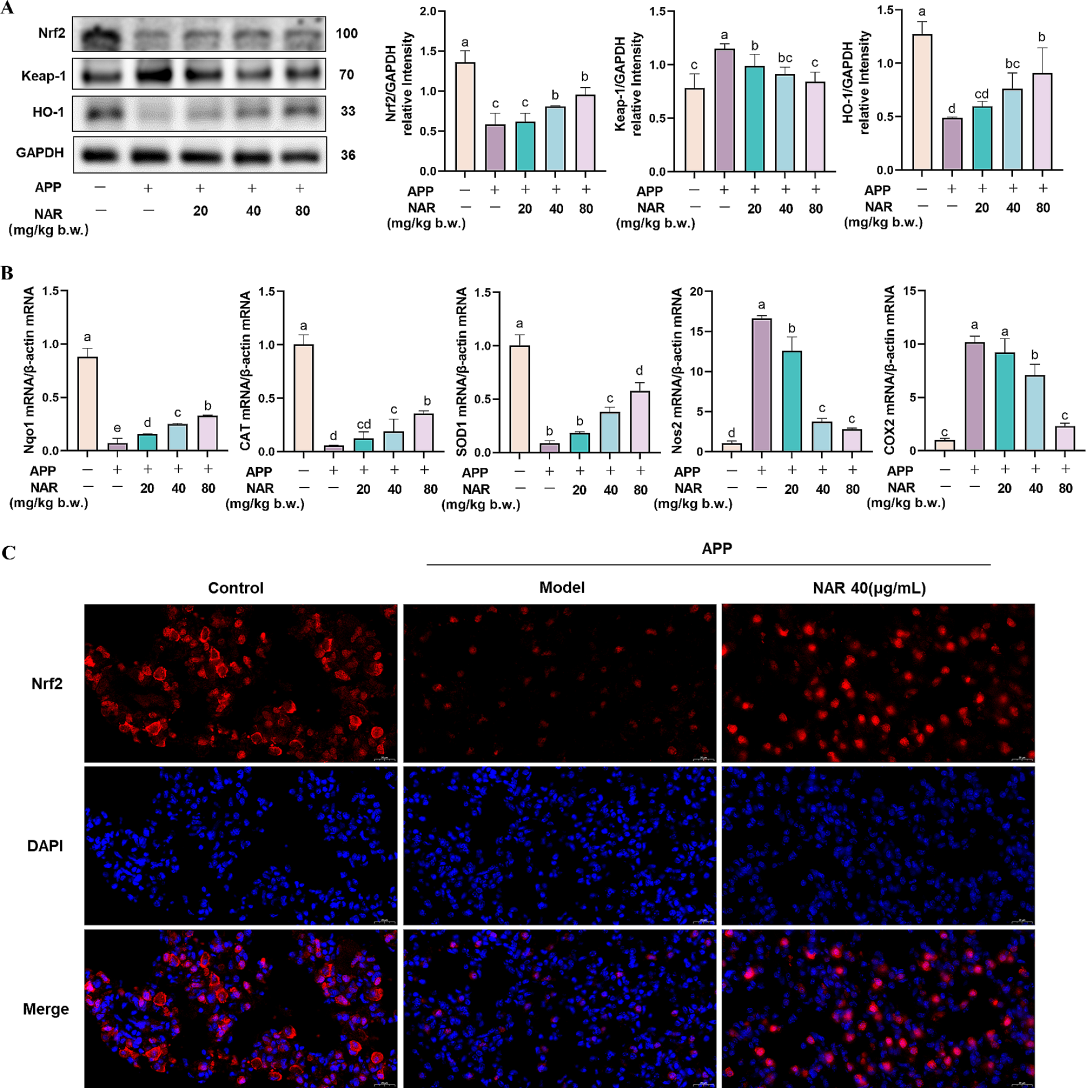
Regulatory effects of NAR on the Nrf2 signalling pathway in the lungs of APP-induced mice. (A) Effects of NAR on the expression of Nrf2, Keap-1, and HO-1 proteins in the lungs of APP-induced mice; (B) Effects of NAR on the face of critical enzymes in the Nrf2 signalling pathway in the lungs of APP-induced mice; (C) Effects of NAR on translocation of Nrf2 protein. Different lowercase letters on the error bars indicate statistically significant differences (*P* < 0.05), results are from one of three independent experiments (*n* = 3)

After APP infection, the excessive inflammatory response attenuates the body’s antioxidant capacity, which induces oxidative stress, aggravating pathological lung damage. Therefore, we tested the anti-oxidative stress capacity of NAR. It was found that the expression of antioxidant proteins Nrf2 and HO-1 was significantly decreased. By contrast, the expression of pro-oxidant protein Keap-1 was increased dramatically in mouse lungs after APP infection (*P <* 0.05). NAR intervention increased the expression of Nrf2 and HO-1 and decreased the expression of Keap-1 (Fig. 4A). Immediately after that, we examined the relative expression of antioxidant enzymes Nqo1, CAT, SOD1, and oxidative damage factors NOS2 and COX2 mRNA in the lung tissues of mice and found that compared with the control group, the mRNA expression levels of Nqo1, CAT, and SOD1 were significantly decreased after APP infection.

In comparison, the mRNA expression levels of NOS2 and COX2 were significantly increased. NAR intervention regressed the expression levels of Nqo1, CAT, and SOD1 and down-regulated the decreased expression levels of NOS2 and COX2(Fig. 4B). Therefore, the results indicated that NAR could promote the activation of the Nrf2 signalling pathway and exert anti-inflammatory effects by inhibiting oxidative stress. Further, immunofluorescence results showed that under normal conditions, Nrf2 exists in the cytoplasm, the total amount of Nrf2 was depleted after APP stimulation, and some of it underwent nuclear translocation into the nucleus to participate in the regulation of antioxidant activity. However, NAR intervention was able to increase the Nrf2 content and promote nuclear translocation significantly (Fig. 4C). Therefore, NAR can encourage the activation of the Nrf2 signalling pathway and thus inhibit APP-induced oxidative stress injury in mouse lungs.

### Effect of NAR on the proliferation of PAMs and secretion of inflammatory factors

**Fig. 5:**
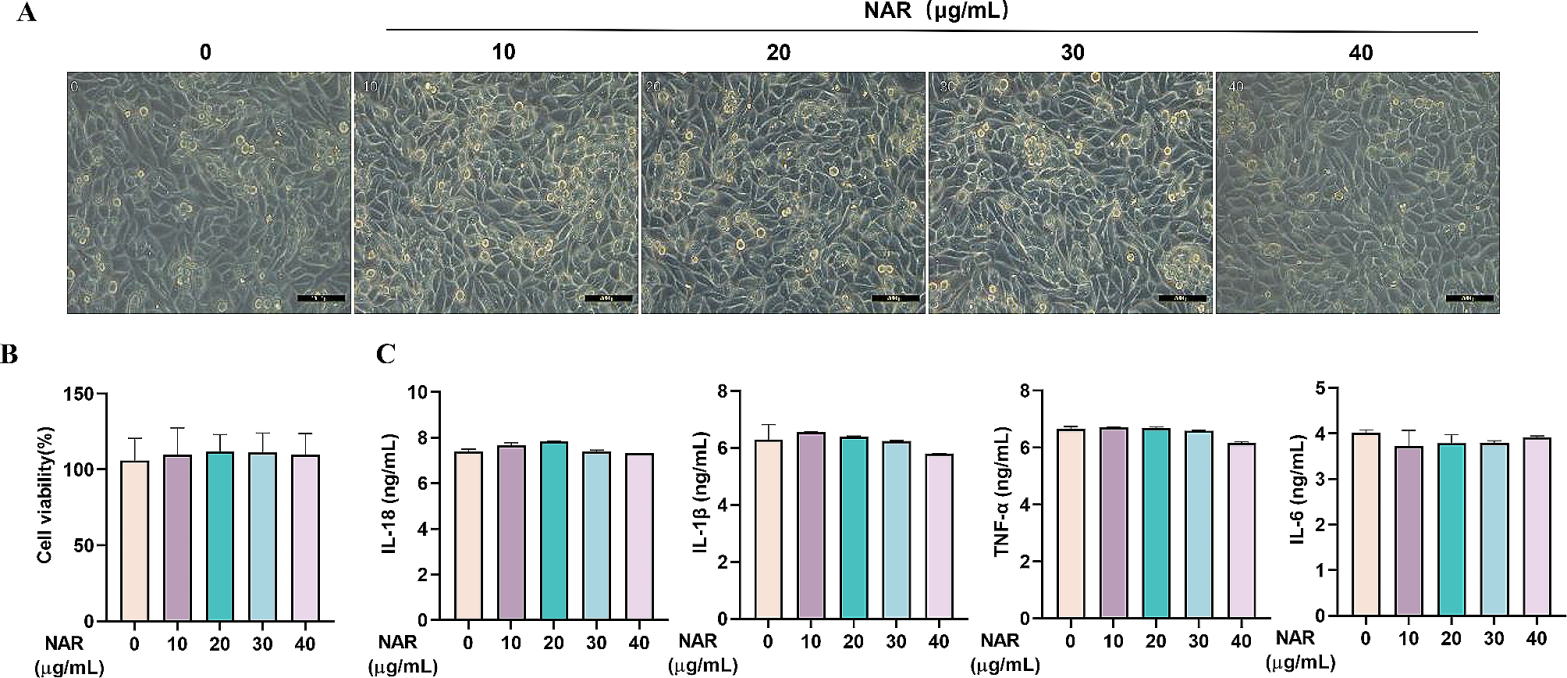
Effect of NAR on proliferation of PAMs as well as secretion of inflammatory factors. (A-B) Effect of NAR on the cellular activity of PAMs, results are from one of eight independent experiments (*n* = 8); (C) Effect of NAR on the secretion of IL-18, IL-1β, IL-6, and TNF-α in PAMs, results are from one of three independent experiments (*n* = 3). Different lowercase letters on the error bars indicate statistically significant differences (*P* < 0.05)

To determine the safe concentration range of NAR in the in vitro assay, we verified the effect of NAR at different concentrations (10, 20, 30, or 40 µg/mL) on cell activity. As observed by microscopic examination, the cell morphology was intact after co-incubation of NAR with PAMs, and no swelling, fracture, shrinkage, or extravasation of cell contents was observed (Fig. 5A). Meanwhile, the proliferative ability of NAR on PAMs was examined by CCK-8 assay. It was found that there was no significant effect on the proliferation of PAMs with the increase of the administered concentration, suggesting that, in the concentration range of 40 µg/mL, NAR was not potentially cytotoxic to PAMs (Fig. 5B). Immediately after that, we determined the effects of different concentrations of NAR on the secretion of inflammatory factors in PAMs by ELISA kit, and the results showed that NAR administration did not affect the secretion of inflammatory factors, such as IL-18, IL-1β, TNF-α, and IL-6, in the cells (Fig. 5C). In conclusion, NAR did not affect the proliferation of PAMs and the secretion of inflammatory factors in the concentration range of 40 µg/mL, which provides a basis for the selection of the concentration of NAR in the subsequent experiments.

### NAR inhibits inflammatory responses in PAMs by modulating the MAPK/NF-κB signalling pathway

**Fig. 6:**
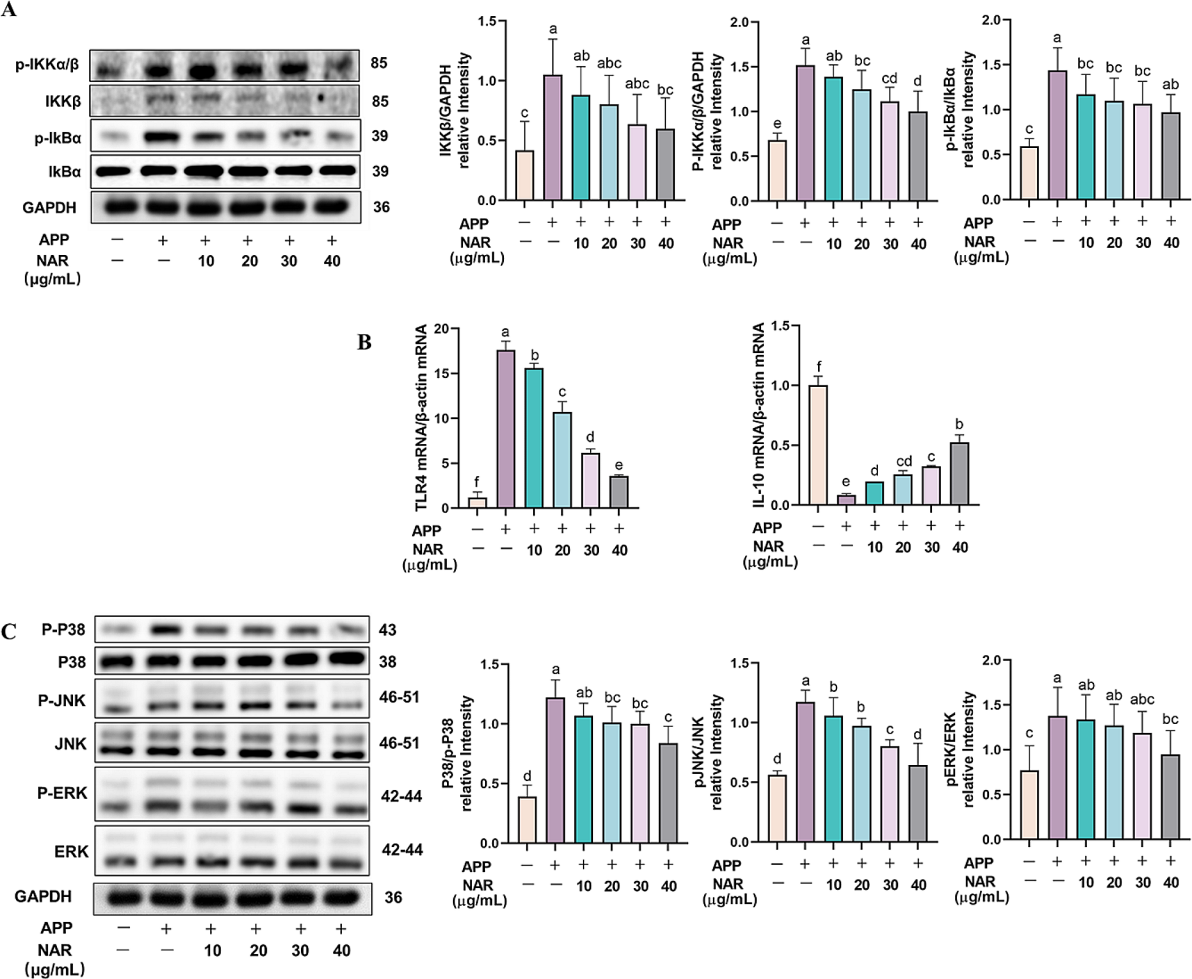
Inhibitory effect of NAR on MAPK/NF-κB signalling pathway.(A) Effect of NAR on NF-κB signalling pathway-related proteins in APP-induced PAMs; (B) Effect of NAR on TLR4 and IL-10 mRNA expression in APP-induced PAMs; (C) Effect of NAR on MAPK signalling pathway-related proteins in APP-induced PAMs. Different lowercase letters on the error bars indicate statistically significant differences (*P* < 0.05), results are from one of three independent experiments (*n* = 3)

PAMs exert phagocytic and antigen-presenting roles in porcine lungs and are a vital defense barrier for pig innate immunity. To demonstrate the anti-inflammatory effects of NAR in PAMs, we first examined the effects of NAR on the NF-κB signalling pathway. Compared with the control group, APP infection promoted the phosphorylation of IKKβ and IκBα proteins in cells. NAR intervention significantly reduced the phosphorylation level of these critical proteins(Fig. 6A). Meanwhile, we found that the expression of TLR4 mRNA in APP-infected cells increased dramatically while the inflammation-suppressing factor IL-10 decreased significantly.

On the contrary, NAR intervention was able to inhibit the mRNA expression of TLR4 and promote IL-10, which in a dose-dependent manner (Fig. 6B). Further, we examined the effect of NAR on the MAPK signalling pathway, which showed the phosphorylation of P38, JNK, and ERK were increased after APP infection compared to the control group, and the phenomenon can be significantly reversed by NAR intervention(*P <* 0.05) (Fig. 6C). In summary, NAR can exert anti-inflammatory effects by inhibiting the over-activation of the MAPK/NF-κB signalling pathway.

### NAR inhibits inflammatory responses in PAMs by modulating the Nrf2 signalling pathway

**Fig. 7:**
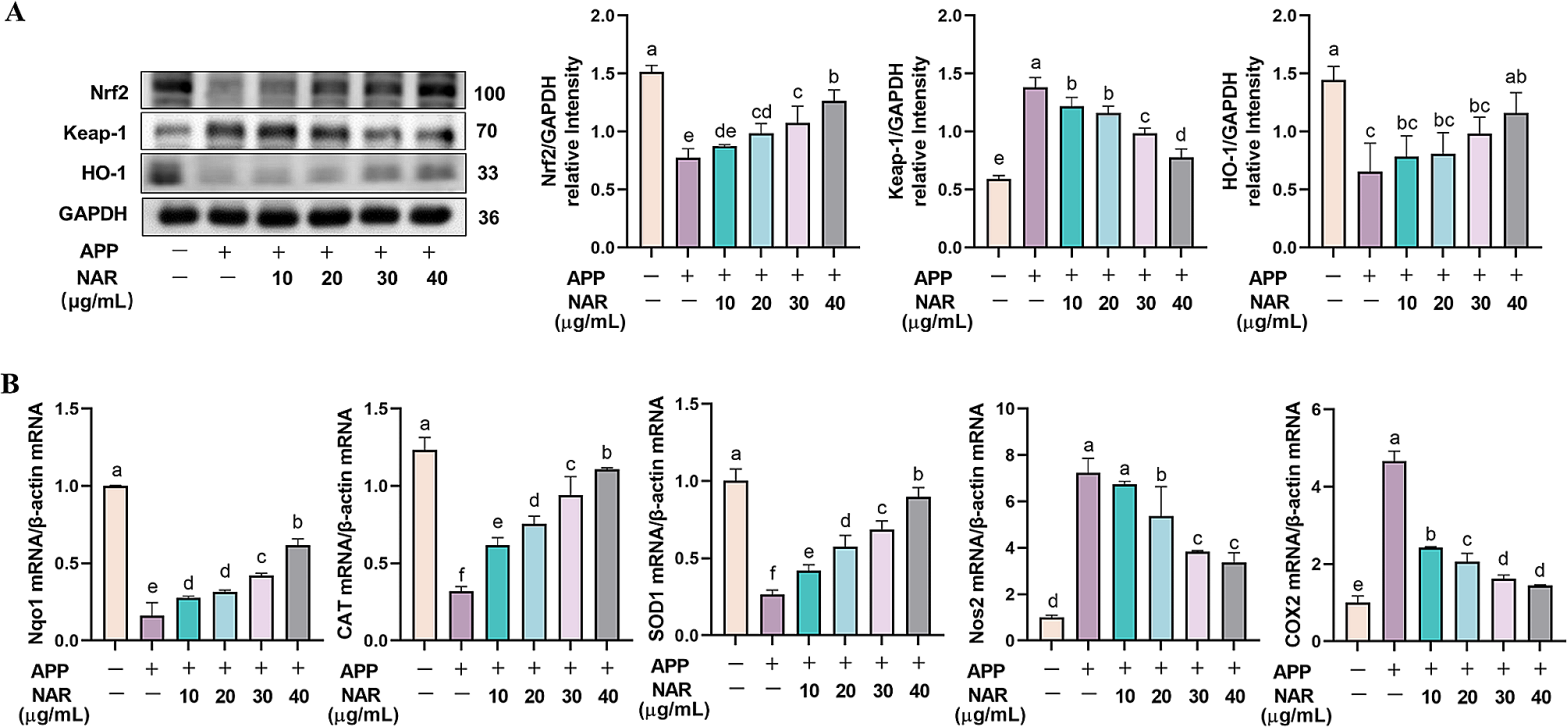
Inhibitory effect of NAR on Nrf2 signalling pathway. (A) Effect of NAR on Nfr2 signalling pathway-related proteins in APP-induced PAMs; (B) Effect of NAR on Nfr2 signalling pathway-related key factors in APP-induced PAMs. Different lowercase letters on the error bars indicate statistically significant differences (*P* < 0.05), results are from one of three independent experiments (*n* = 3)

To further elucidate the anti-inflammatory effect of NAR, we explored the antioxidant capacity of NAR in PAMs. The results showed that compared to the control group, the protein expression of both Nrf2 and HO-1 in PAMs after APP infection decreased while the Keap-1 was increased significantly. The intervention of NAR was able to dramatically elevate the Nrf2 and HO-1 and reduce Keap-1protein expression in cells (Fig. 7A). In addition to this, critical factors in the Nrf2 signalling pathway were also examined. It was found that the gene expression of the antioxidant enzymes Nqo1, CAT, and SOD1 was significantly elevated in cells after APP stimulation compared with that of the control group (*P <* 0.05). In contrast, the gene expression of the oxidative damage factors NOS2 and COX2 was decreased. In the NAR administration group, the mRNA expression of Nqo1, CAT, and SOD1 increased (*P <* 0.05). At the same time, the NOS2 and COX2 were significantly decreased in a dose-dependent manner (Fig. 7B). The results indicated that NAR could play an anti-inflammatory role by activating the Nrf2 pathway, inducing an increase in the expression of antioxidant enzymes, thereby reducing the cellular oxidative stress damage caused by APP.

## Discussion

NAR is widely found in the peels of citrus fruits such as grapefruit, tangerines and oranges, yet these peels are common agricultural wastes. Therefore, it is necessary to estimate the value of these agricultural by-products for their applications. According to the literature, NAR has a wide range of pharmacological activities, such as anti-inflammatory [20], antioxidant [21], anti-apoptotic [22], and anti-tumor [22]. In clinical practice, NAR has a significant restorative effect on respiratory inflammatory injury, which can significantly reduce sputum secretion and inflammatory infiltration in the lungs, decrease the proliferation of cuprocyte, and reduce mucus secretion, and so on [23, 24]. . In this study, we focused on the protective effect of NAR on APP-induced inflammatory lung injury to provide a theoretical basis for the development of natural drugs to prevent this disease.

The pathogenic mechanism of APP is mainly through the secretion of various virulence factors that adhere to the alveolar epithelium and continuously stimulate PAMs. Because PAMs are an essential immune barrier in the lungs [25], pattern-recognition receptors (PRRs) on the cell surface and intracellular activate the MAPK/NF-κB signalling pathway by recognizing the virulence factors, which activate innate immunityand promote the inflammatory factor release [26, 27]. When the organism is subjected to sustained strong stimuli and inflammatory reactions, the immune balance of the organism is disrupted, leading to a series of cytokine cascade responses that promote the massive aggregation of NEs in the alveoli, causing elevated WBCs and CRP in the peripheral blood as well as inflammatory injury to lung tissues caused by pulmonary edema and inflammatory infiltration. At the same time, due to platelet clumping in the lung tissue, the capillaries rupture leading to further necrosis of the lung tissue [28]. Our results showed that the lung tissues of mice in the model group also showed lesions of typical symptoms, such as congested alveolar capillaries with a large number of erythrocytes, WBCs, NEs, as well as fibrinous thrombus formation in the blood vessels. A fibrous mesh was formed in the alveolar lumen and connective tissue proliferation was observed. The results showed that the number of leukocytes and NE in the peripheral blood of APP-infected mice was reduced after NAR intervention, a result consistent with our previous findings that NAR suppressed the increase in the number of NE in the alveolar lavage fluid induced by APP [14]. Therefore, NAR significantly alleviated the above symptoms of APP-induced lung injury in mice.

Previous studies have found that APP can induce an inflammatory response in vivo mainly through activation of the MAPK/NF-κB signaling pathway [29], and this phenomenon is also verified in our experiment. In this study, we found that NAR can reduce the expression levels of p-IKKα/β, IKKβ, and p-IKKα at the protein level, inhibit the expression of TLR4 at the gene level, and increase the expression of anti-inflammatory factor IL-10. MAPK is a serine/threonine protein kinase that is widely expressed and has a very critical role in inflammation and its signalling cascade consists of three major branches, P38, c-Jun N-terminal kinase (JNK) and extracellular signal-related kinases (ERK) [17]. Therefore, in the study, it was found that NAR was able to reduce the phosphorylation levels of P38, JNK and ERK in a dose-dependent manner. It is suggested that NAR can play an anti-inflammatory role by inhibiting the activation of the MAPK/NF-κB signalling pathway, inhibiting the secretion of pro-inflammatory factors, and increasing the secretion of anti-inflammatory factors after APP infection.

In recent years, relevant studies have found that APP causes excessive inflammation in the body, which triggers the emergence of oxidative stress damage [18]. Nrf2 is an crucial transcription factor related to oxidative stress of the body. Under normal circumstances, Nrf2 and Keap-1 bind and exist universally in the cytoplasm. After oxidative stress, electrotropic metabolites inhibit the activity of Keap1 complex, promote the translocation of Nrf2 into the nucleus, induce the downstream expression of HO-1, enhance the expression of antioxidant enzymes such as Gpx and SOD, and play the role of antioxidants. Therefore, we also explored the antioxidant effect of NAR in the present study and found that NAR could increase the protein content of Nrf2 and HO-1 and decrease the protein content of Keap-1 through *in vivo/in vitro* studies. It also proved that NAR was able to increase the transcript levels of antioxidant enzymes Nqo1, CAT, and SOD1 and inhibited the transcript levels of oxidative damage factors NOS2 COX2, thus improving the antioxidant capacity of the organism. It was found by immunofluorescent labeling of Nrf2 that it was prevalent in the control group in the cytoplasm, and Nrf2 entered the nucleus after APP stimulation. In contrast, NAR intervention increased the amount of Nrf2 in the cell and facilitated its entry into the nucleus. Taken together, NAR can alleviate lung injury induced by modulating anti-inflammatory and antioxidant dual signalling after APP infection.

## Conclusions

**Fig. 8:**
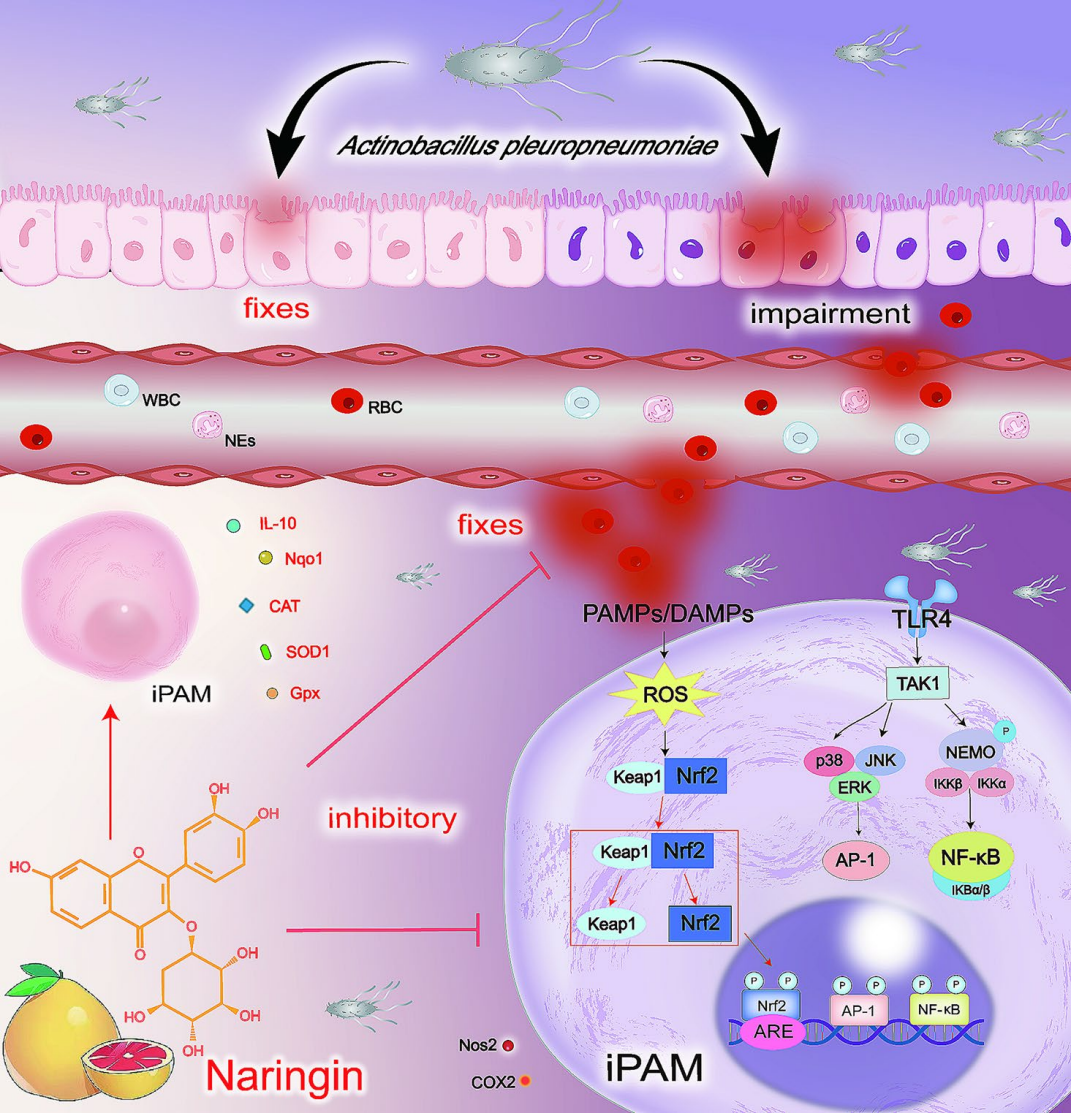
NAR alleviates APP-induced pneumonia through dual anti-inflammatory and antioxidant pathways. When APP enters the organism, it adheres to and deposits in the alveolar epithelium. It continuously stimulates PAMs, which activates the activation of the MAPK/NF-κB signalling pathway and thus promotes the release of inflammatory factors, causing inflammation to be generated. In contrast, excessive inflammation triggers oxidative stress injury, which enables the accumulation of large numbers of NEs and WBCs in peripheral blood, as well as the inflammatory injury of lung tissue caused by pulmonary edema and inflammatory infiltration. At the same time, it induced platelet aggregation in lung tissue, which led to the rupture of capillaries, leading to necrosis of lung tissue. It was found that NAR could exert anti-inflammatory effects by inhibiting the activation of the MAPK/NF-κB signalling pathway and antioxidant effects by activating the Nrf2 signalling pathway, thus alleviating the APP-induced lung injury

In summary, the present study demonstrated that NAR can attenuate APP-induced lung injury through anti-inflammatory and antioxidant pathways. The mechanism of action may be dependent on inhibiting the MAPK/NF-κB inflammatory signalling pathway and promoting the activation of the Nrf2 antioxidant signalling pathway. Therefore, our study elucidated the protective mechanism of NAR against APP, aiming to provide new options for the development of alternative therapies (Fig. 8).

### Electronic supplementary material

Below is the link to the electronic supplementary material.

Supplementary Material 1

Supplementary Material 2

## Data Availability

The datasets used and/or analyzed during the current study are available from the corresponding author upon reasonable request.

## Abbreviations

NAR: Naringin
APP: *Actinobacillus pleuropneumoniae*
NF-κB: nuclear factor kappa-B
Nrf2: nucleafactorerythroid-2-relatedfactor2
MAPK: mitogen-activated protein kinase family
TLR4: Toll-likereceptor4
NEs: neutrophils
WBCs: white blood cells
IL-10: interleukin-10
HO-1: Heme oxygenase-1
Keap-1: Recombinant kelch like ech associated protein 1
NQO1: NADH Quinone Oxidoreductase 1
CAT: Catalase
SOD1: Superoxide Dismutase1
Nos2: Nitric Oxide Synthase 2
COX2: Cytochrome Oxidase Subunit 2
Gpx1: Glutathione peroxidase 1
