# Supplementary material for: Naringin attenuates Actinobacillus pleuropneumoniae-induced acute lung injury via MAPK/NF-κB and Keap1/Nrf2/HO-1 pathway
Source: BMC Vet Res. 2024 May 17;20:204. doi: 10.1186/s12917-024-04055-2 (PMC11100192; doi:10.1186/s12917-024-04055-2)

1. The Marker we used was Purchase from EpiZyme Biological Co.，item number is WJ102.


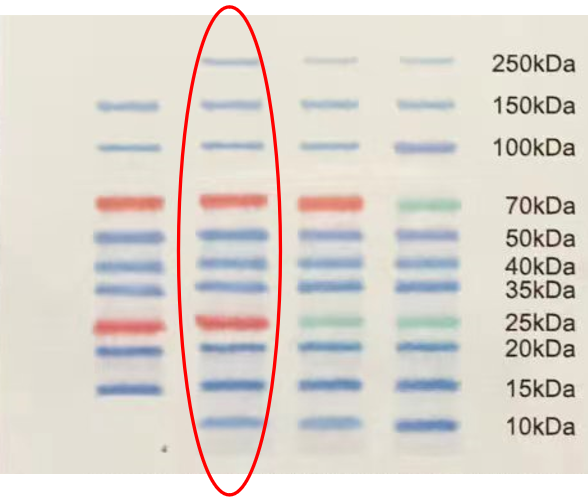


1. Experimental apparatus to WB

Instrument Model: GeneGnome Purchase from Gene Company Limited


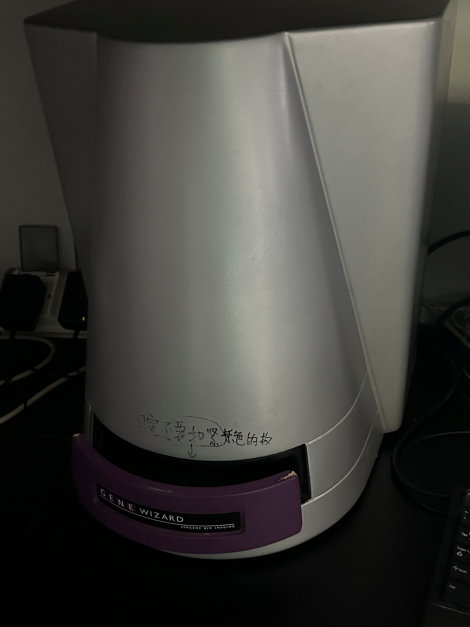

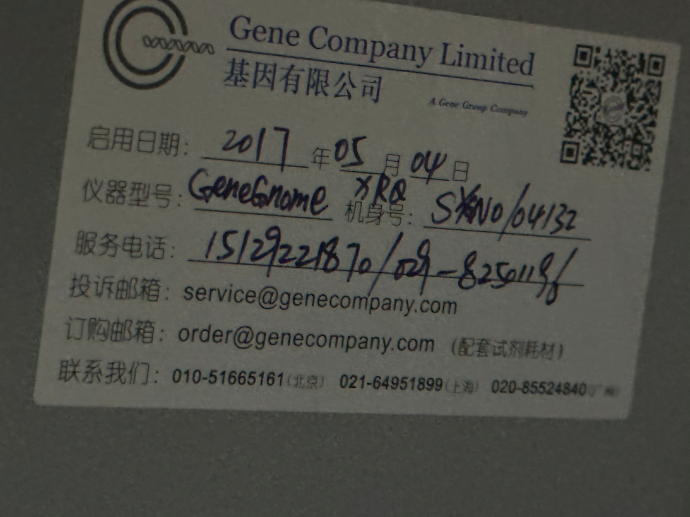


1. After taking photos with this instrument, there are three ways to save the photos, and we often choose the second way to keep them. But sometimes, we may encounter overexposure, which can cause the mark's imprint to become very faint. In this case, we will determine the position of the mark based on the first save mode.


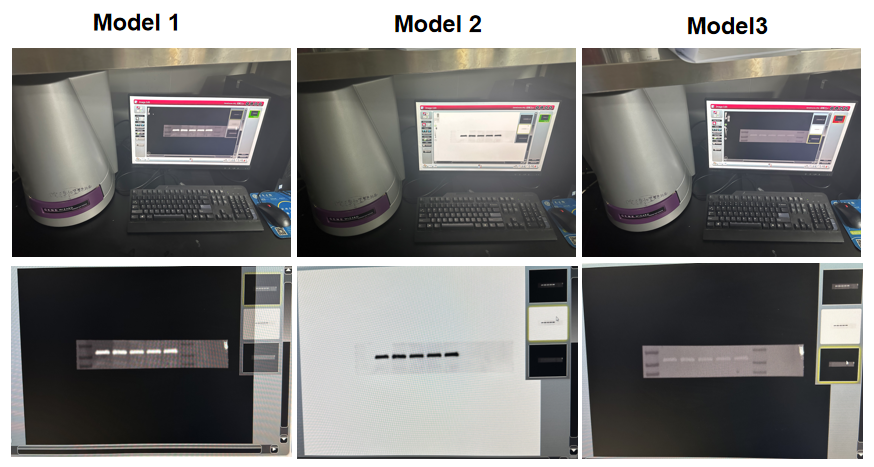

Supplement: Supplementary file 1 — Supplementary Material 1 [file 12917_2024_4055_MOESM1_ESM.docx]
