# Supplementary figures and images for: Naringin attenuates Actinobacillus pleuropneumoniae-induced acute lung injury via MAPK/NF-κB and Keap1/Nrf2/HO-1 pathway
Source: BMC Vet Res. 2024 May 17;20:204. doi: 10.1186/s12917-024-04055-2 (PMC11100192; doi:10.1186/s12917-024-04055-2)

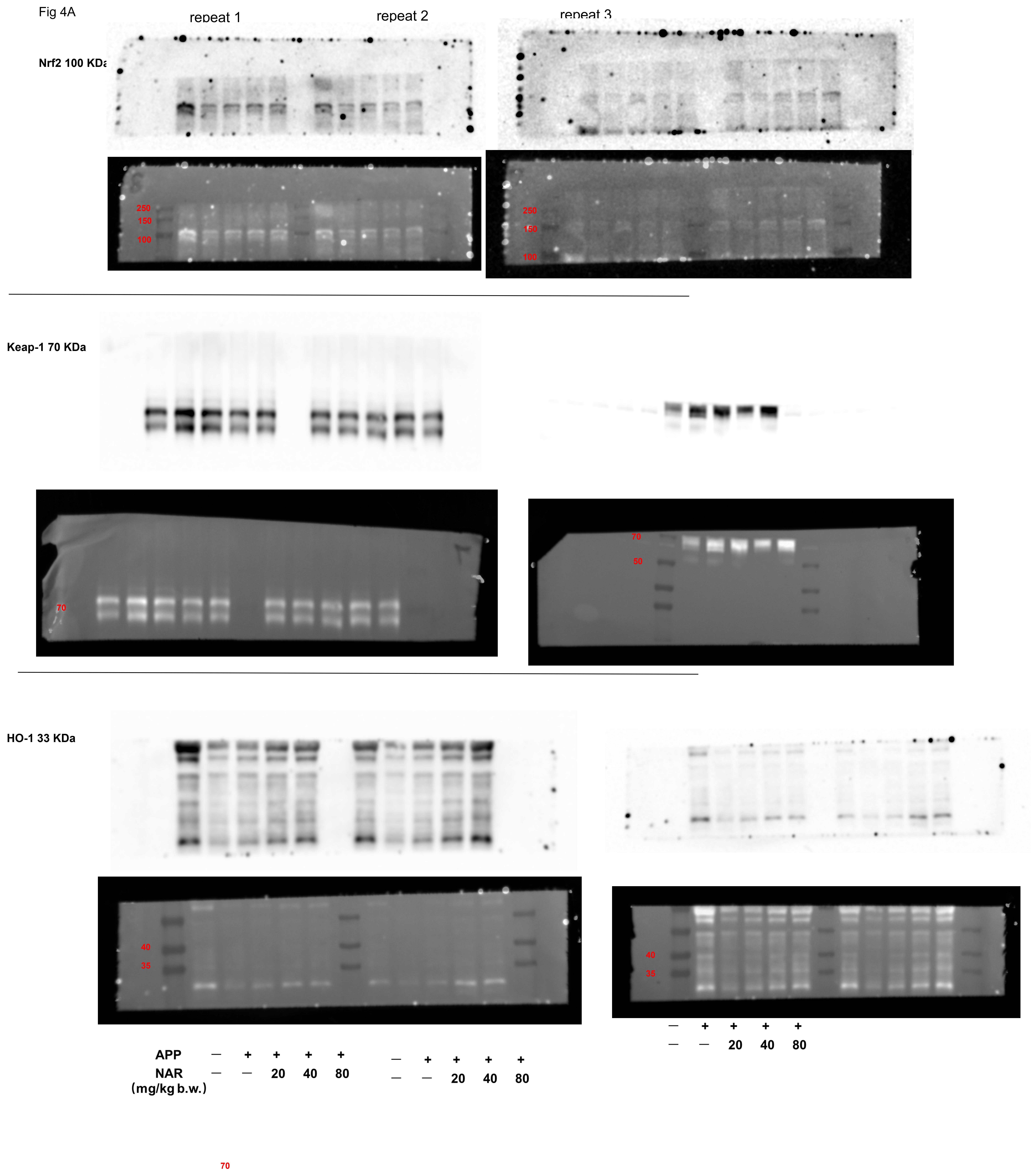

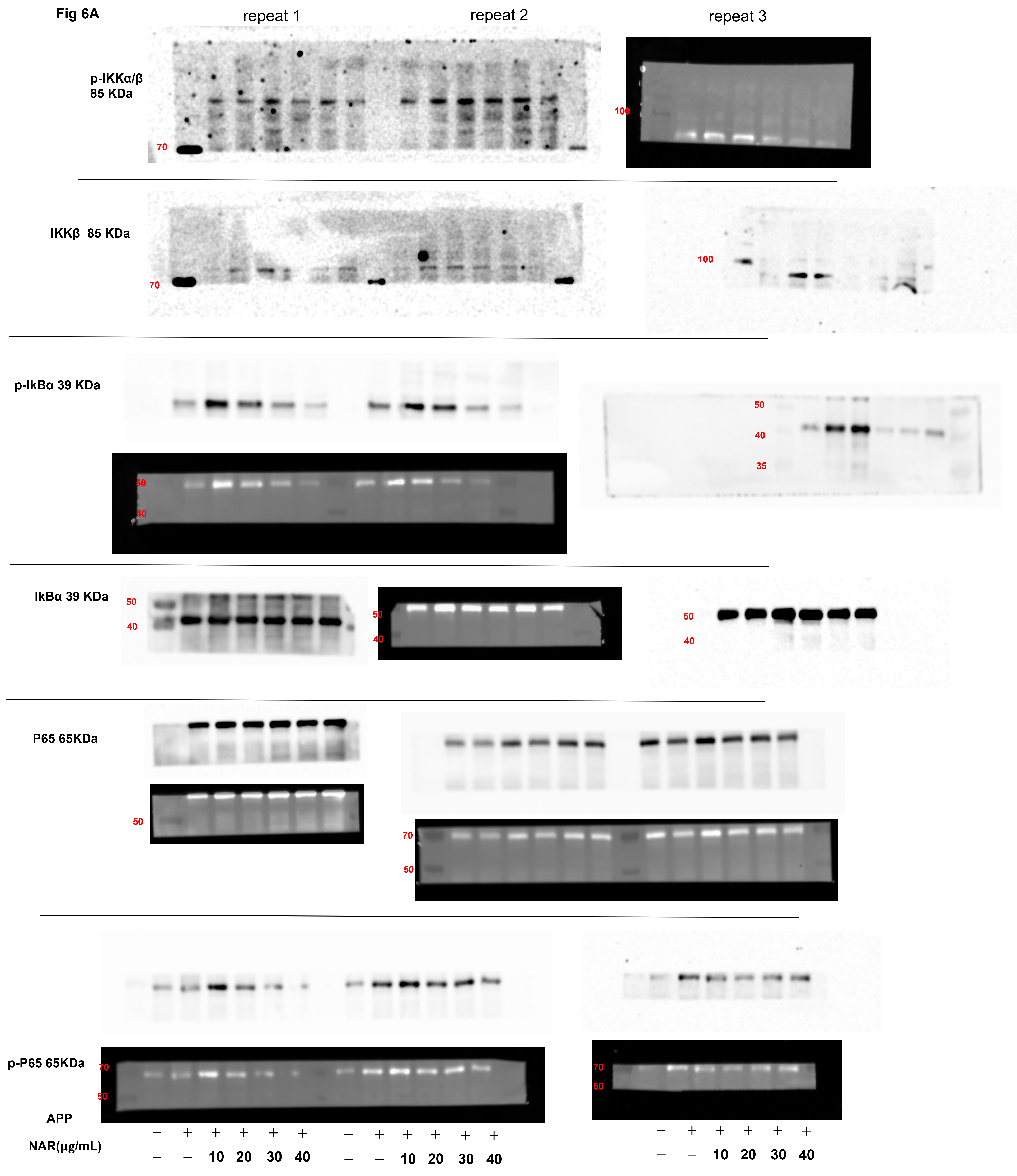




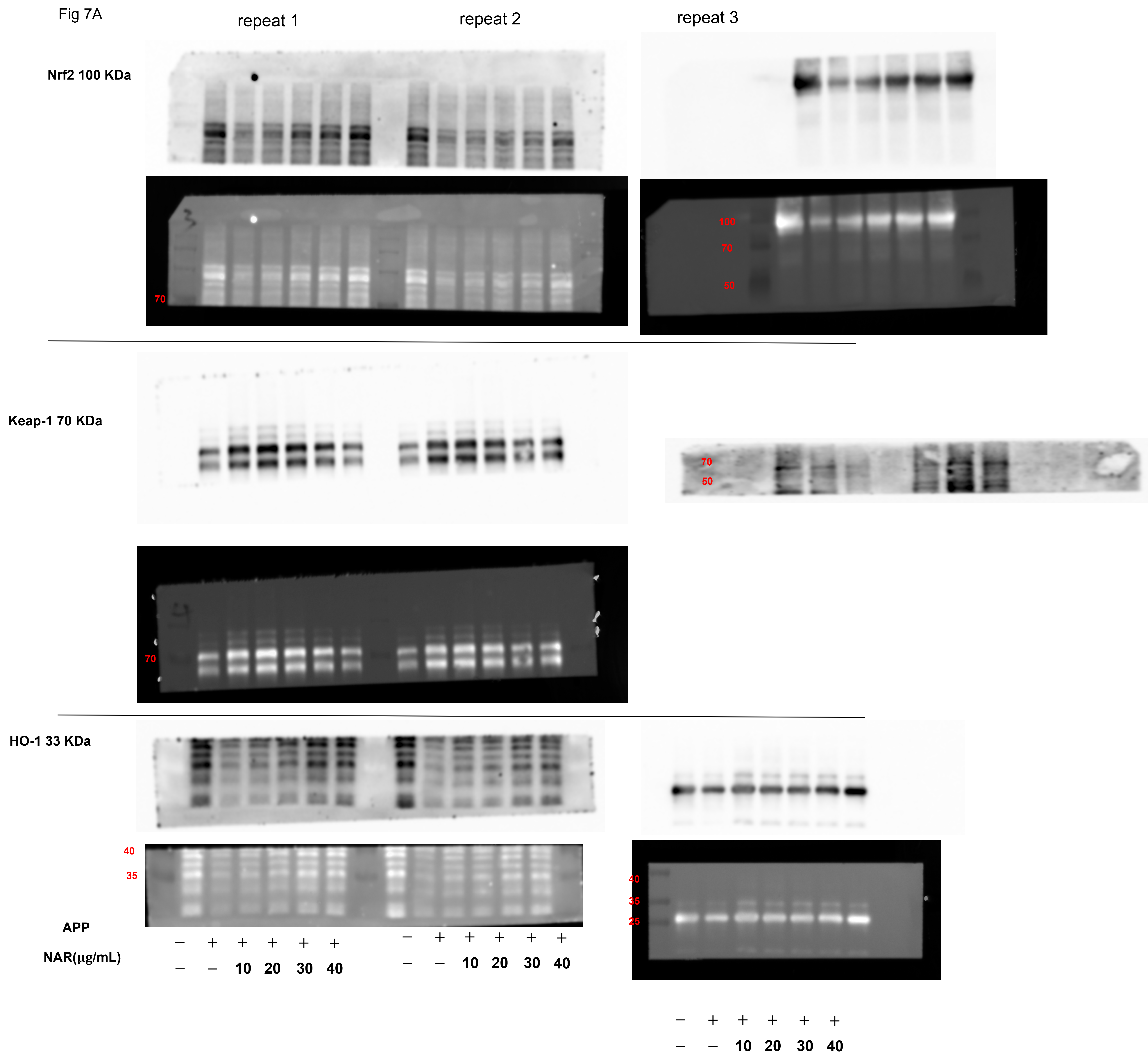

Supplement: Supplementary file 2 — Supplementary Material 2 [file 12917_2024_4055_MOESM2_ESM.doc]
